# Standardized protocol of blood pressure measurement and quality control program for the Korea National Health and Nutrition Examination Survey

**DOI:** 10.1186/s40885-023-00252-7

**Published:** 2023-10-12

**Authors:** Hack-Lyoung Kim, Sang Min Park, In Jeong Cho, Yu-Mi Kim, Dae-Hee Kim, Sung Hye Kim, Kwang-Il Kim, Ki-Chul Sung, Sang-Hyun Ihm, Jinho Shin, Yoonjung Kim, Kyungwon Oh, Eun Mi Lee, Quality control team of blood pressure measurements for the Korea National Health, Nutrition Examination Survey (KNHANES)

**Affiliations:** 1 Division of Cardiology, Department of Internal Medicine, Boramae Medical Center, Seoul National University College of Medicine, Seoul, Republic of Korea; 2 Divison of Cardiology, Department of Internal Medicine, Nowon Eulji Medical Center, Eulji University College of Medicine, Seoul, Republic of Korea; 3 Division of Cardiology, Department of Internal Medicine, Ewha Womans University Seoul Hospital, Ewha Womans University College of Medicine, Seoul, Republic of Korea; 4 Department of Preventive, College of Medicine, Hanyang University, Seoul, Republic of Korea; 5 Hanyang University School of Public Health, Seoul, Republic of Korea; 6 Division of Cardiology, Department of Internal Medicine, Asan Medical Center, University of Ulsan College of Medicine, Seoul, Republic of Korea; 7 Department of Pediatrics, CHA Bundang Medical Center, CHA University, Seongnam, Republic of Korea; 8 Division of Geriatrics, Department of Internal Medicine, Seoul National University Bundang Hospital, Seoul National University College of Medicine, Seongnam, Republic of Korea; 9 Division of Cardiology, Department of Internal Medicine, Kangbuk Samsung Hospital, Sungkyunkwan University School of Medicine, Seoul, Republic of Korea; 10 Division of Cardiology, Department of Internal Medicine, College of Medicine, Bucheon St. Mary’s Hospital, The Catholic University of Korea, Bucheon, Republic of Korea; 11 Division of Cardiology, Department of Internal Medicine, College of Medicine, Hanyang University, Seoul, Republic of Korea; 12 Division of Health and Nutrition Survey and Analysis, Bureau of Chronic Disease Prevention and Control, Korea Disease Control and Prevention Agency, Cheongju, Republic of Korea; 13 Division of Cardiology, Department of Internal Medicine, Wonkwang University Sanbon Hospital, Gunpo, Gyeonggi-do 15865 Republic of Korea

**Keywords:** Health survey, Blood pressure, Sphygmomanometers, Oscillometry, Standardization, Quality controls

## Abstract

Accurate blood pressure (BP) measurement is crucial for hypertension detection and management. The Korea National Health and Nutrition Examination Survey (KNHANES) assesses the health of Koreans using representative cross-sectional data. BP measurements were historically done with mercury sphygmomanometers for participants aged ≥10 years. However, KNHANES transitioned to Greenlight 300^TM^ (mercury-free auscultatory device) in 2020 for participants aged ≥6 years and used dual devices (Microlife WatchBP Office AFIB and Greenlight) in 2021-2022. To ensure consistency, KNHANES will adopt Microlife as the unified BP device with Greenlight for device validation from 2023. Under the new protocol, participants aged ≥6 years will have their BP measured three times at 30-second intervals after a 5-minute rest under ambient temperature (20-25℃) and noise ≤65 dB. The average of the 2nd and 3rd readings will be used as the representative BP value. The quality control (QC) program involves four trained examiners passing the “quality control and assurance of BP measurement program” three times annually, and undergoing “video monitoring of weekly calibration process” once a year. Additionally, the QC team will conduct “on-site evaluations of BP measurement” at mobile examination centers three times a year. A Five-Step QC process for BP devices was also developed. This document outlines the standardized BP measurement protocol and rigorous QC program in KNHANES, aiming to ensure accurate and reliable BP data for epidemiological research and public health policymaking in South Korea.

**Graphical Abstract:**

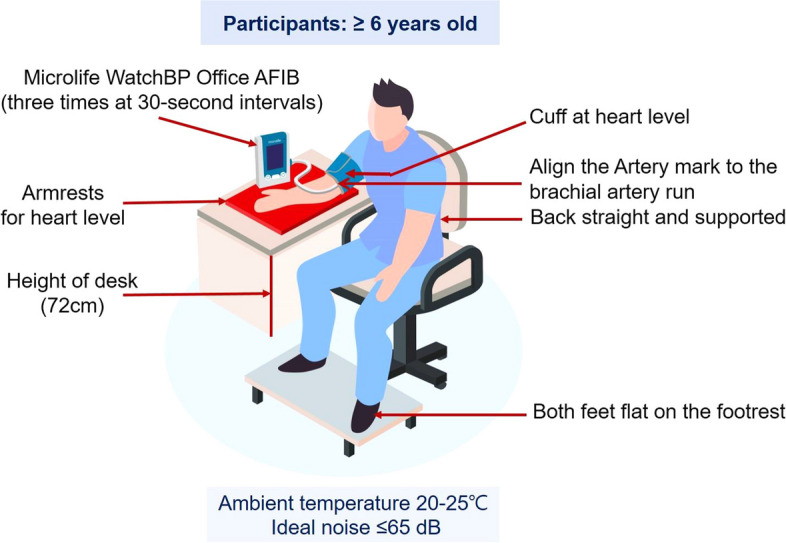

**Supplementary Information:**

The online version contains supplementary material available at 10.1186/s40885-023-00252-7.

## Introduction

Hypertension is the major cause of cardiovascular disease and premature death worldwide. Its prevalence was approximately 30% of the adult Korean population [1]. Therefore, to reduce major public concern, accurate blood pressure (BP) measurement is essential for early detection and proper management of hypertension. The Korea National Health and Nutrition Examination Survey (KHNANES) is a representative population-based cross-sectional survey designed to assess the health and nutritional status of Korean citizens conducted by the Korea Disease Control and Prevention Agency (KDCA) [2]. In KNHANES, from 1998, four examiners (trained nurses) measured BP using mercury sphygmomanometers (MSs) among participants aged ten years or more in four mobile examination centers (MECs) [3]. BP values can be greatly influenced by a variety of factors, including those related to the subject, the examiner, and the surrounding environment [4]. Thus, it is essential to minimize these measurement errors [5]. Furthermore, the KNHANES is a representative database of the Korean population, widely utilized for computing various statistics and setting indicators. Inappropriate BP measurements or systematic errors within the framework of KNHANES can lead to significant changes in fundamental values related to hypertension. Therefore, ensuring accurate BP measurement in KNHANES is of the utmost importance. To maintain this accuracy, KNHANES adheres to a standardized protocol for BP measurement and routinely conducts quality control (QC) and assurance projects pertaining to BP measurements. However, MSs have been banned in Korea because of Minamata Convention on Mercury for mercury toxicities. Thus, MSs should be replaced with mercury-free sphygmomanometers for BP measurement [6].

Mercury-free sphygmomanometers are divided into two types according to measurement techniques [6]: electronic auscultatory devices (ADs, Korotkoff method) or oscillometric devices (ODs, automated electronic devices). KDCA conducted several comparative studies in subjects participating in KNHANES to find the proper mercury-free BP device. Choi et al*.* [7] indirectly compared the BP difference of HEM-907^®^ (OD, Omron, Kyoto, Japan) versus MS and Greenlight 300^TM^ (AD, Greenlight, Accoson, Essex, United Kingdom) versus MS. They showed that the Greenlight may be a good alternative to the MS, and the HEM has good accuracy in systolic BP, but it was inferior to the Greenlight due to the measurement error in diastolic BP. Also, Greenlight fulfills the accuracy criteria permitted by the Universal protocol of BP device validation [8]. Moreover, Kim et al*.* [9] reported that Greenlight is a suitable alternative to an MS as a reference standard for BP device validation. Recently, Lee et al. [10] evaluated the validity of Microlife WatchBP Office AFIB^®^ (OD, Microlife, Microlife AG, Taiwan), and they reported that it had similar accuracy in systolic and diastolic BP against Greenlight in subjects participating in KNHANES.

Based on the studies above [7, 9, 10] and intense discussion collaborated with the Korean Society of Hypertension, KDCA decided to measure BP for KNHANES with Greenlight from participants aged six and over in 2020 [11]. In 2021-2022, KNHANES measured BP using dual devices [12]: Microlife for adults (≥19 years) and Greenlight for pediatrics (6-18 years) because several guidelines recommend that OD is a screening tool, whereas AD is a confirmatory device for BP measurement for pediatrics [13, 14]. However, considering the difficulties in QC of BP measurement due to using dual devices, KDCA has decided the use Microlife as a unified BP device on participants aged six and over in KNHANES from 2023 [15]. Whereas Greenlight will be used only as a reference device for BP device validation to replace the MS (Table 1).

**Table 1:** BP measuring devices for KNHANES from 1998

Year	1998-2019	2020	2021-2022	≥2023
Devices	Mercury sphygmomanometer	Greenlight (mercury-free AD)	6-18 years: Greenlight ≥19 years: Microlife	Microlife (OD)
Participants	≥aged 10 years	≥aged 6 years		
Place	Four mobile examination centers			

*BP* Blood pressure, *KHNANES* Korea National Health and Nutrition Examination Survey, *Greenlight* Greenlight300^TM^, *Microlife* Microlife WatchBP Office AFIB^®^, *AD* Auscultatory device, *OD* automated oscillometric device

Therefore, in this document, we aim to describe the standardized protocol of BP measurement with Microlife and Greenlight, and their QC and assurance project for KNHANES.

## Part 1. BP measurement using Microlife Watch BP Office AFIB^®^

KNHANES will obtain BP values using the oscillometric device (Microlife WatchBP Office AFIB^®^) three times at 30-second intervals after a five-minute rest on participants aged six and over from 2023 under ideal ambient temperature 20-25 ℃ and ideal noise ≤65 dB in 4 MECs. The representative BP value is the average of the 2^nd^ and 3^rd^ BP readings.

### Participants

KNHANES will obtain BP values on participants aged six and over. Individuals with rashes, open wounds, weakness, splints, edema, hematomas, arteriovenous fistulas for dialysis on both arms, are excluded from the measurement. In women with axillary node biopsy/surgery or radical mastectomy at one side, the contralateral arm is used for BP measurement. Women who underwent surgery on both sides were excluded from the BP measurement. Participants with an arm circumference (AC) of <14cm, or >52cm were excluded from BP measurement.

### Device details

**Fig. 1:**
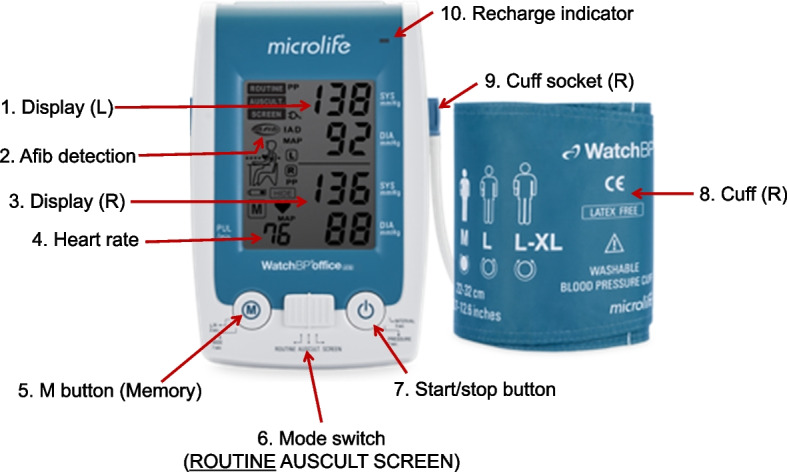
Description of Microlife. Microlife, Microlife WatchBP Office AFIB^®^; L, left arm; R, right arm; Afib, atrial fibrillation; AUSCULT, auscultatory mode; SCREEN, screening mode

**Fig. 2:**
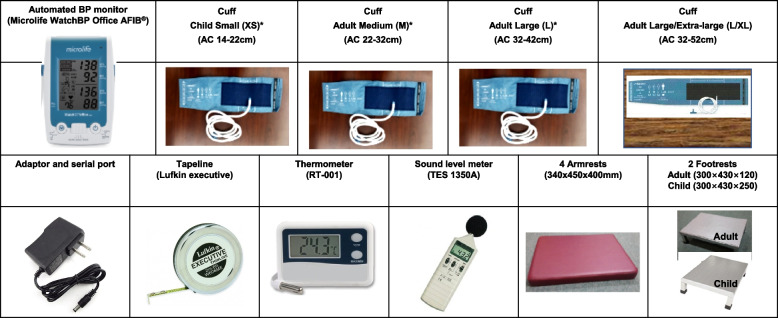
Components of Microlife. ^*^Internationally validated. AC, arm circumference

Device structures are shown in Fig. 1 [16]. Microlife is an oscillometric BP device for the upper arm, which is an internationally validated BP device in adults and children that has passed the standard protocol of the European Society of Hypertension-International Protocol (ESH-IP) [17, 18]. Moreover, this device showed similar systolic and diastolic BP accuracy against the standard reference device of AD (Greenlight) in subjects participating in KNHANES [10]. Microlife has a wide range of cuff sizes according to AC [19] and can accurately measure BP even in atrial fibrillation [20]. In the oscillometric method, BP is measured by detecting the magnitude of pressure oscillation in the cuff over the arterial blood vessel during the period of cuff deflation. When the amplitude of the oscillation wave is the maximum, the cuff pressure of this point estimates the mean arterial pressure. According to predetermined algorithms, the systolic and diastolic BPs are calculated from the measured mean arterial pressure [21]. Thus, the main limitation of the oscillometric method is that it is difficult to control the measurement error because the shape of the oscillation wave varies depending on many factors, such as blood vessel structure, stiffness [22], and AC. Components for BP measurement with Microlife are shown in Fig. 2. Tapeline is used to measure AC, a thermometer to measure temperature, a sound level meter to detect noise from the surrounding environment, armrests to match the arm with the level of the heart, and footrests for adults and children whose feet do not touch the floor.

### Device setting

The default settings of Microlife for BP measurement are as shown in Table 2.

**Table 2:** Default settings of Microlife

**Setting item**	**Set value**
Action mode	ROUTINE mode
Default pressurization setting	Automatic pressurization
Default cuff side	Right arm cuff (R), Switch to left arm cuff (L) for left arm measurement
HIDE mode setting	OFF mode
Measurement numbers	Three consecutive measurements with 3- second intervals in between, but no more than five consecutive measurements

*Microlife* Microlife WatchBP Office AFIB^®^

- ◽ Default setting [12, 16]
  - Select an operation mode, «ROUTINE» Mode
  - Select the right (R) cuff
    - Device is defaulted to the left (L) arm cuff → Press and hold the M button for 3 seconds → Switch the left cuff to the right (R) cuff → Press the Start/Stop button to save the setting.
    - Switch to the right (R) cuff whenever you turn on the power button.
    - Connect the cuff to the device by inserting the cuff connector in the cuff connector socket (right socket for right arm cuff)
  - Set on automated triple measurements with 30-second intervals
    - The device automatically takes three consecutive measurements at default 15-second intervals → Pressing and holding the Start/Stop button for 3 seconds → Press the M Button to adjust the measurement intervals, and be set as 30-second intervals → Press the Start/Stop Button to confirm.
  - Activates HIDE function, “OFF”
    - The Microlife has defaulted features a “HIDE function, ON” to prevent unnecessary elevated BP in patients due to nervousness triggered by visible BP measurements. However, in the KNHANES, it was set as HIDE mode “OFF” feature to facilitate interpretation of BP values and detect errors during BP measurements → Press and hold the M Button for 7 seconds → Press the M Button again to turn the “HIDE OFF” function → Press Start/Stop button to confirm the setting.
- ◽ Display the “Afib” icon to detect atrial fibrillation

The Microlife is designed to detect asymptomatic atrial fibrillation during BP measurements in «ROUTINE» Mode [16]. If atrial fibrillation is detected during each BP measurement, the Afib icon is activated. Microlife’s atrial fibrillation detection sensitivity is 80%, and specificity is 98% [23]. However, a false-positive result may occur in pediatric subjects, so its use is generally recommended for subjects over 65. Children are instructed not to take excessive deep breaths to prevent their posture from moving during BP measurement and to reduce the false positive rate from detecting atrial fibrillation.

### Preparations before BP measurement

- ◽ Wear a disposable gown for the examination

Participants aged 10 years and over wear a disposable gown for the examination.

- ◽ Sitting position

The subject sits on the back of the chair in a stable and comfortable position with back support. The subject should not cross both legs and allow both feet to touch the floor flat. If the subject’s feet do not touch the floor, use a footrest to touch the floor.

- ◽ History taking

Thirty minutes before the measurements, ask questions about cigarette smoking, coffee ingestion, and past history, *etc*., and record the information on the questionnaire survey sheet and KDCA web system. When asking questions during the five-minute rest period, guide them so that they can respond with minimal movement without disturbing their stability.

- ◽ Arm selection

For standardization, the right arm is usually selected for BP measurement. However, if the right arm meets the exclusion criteria, BP is measured on the left arm.

- ◽ Pulse measurement

Pulse measurement is performed to recognize the individual’s basic vital signs and changes in the pulse, such as arrhythmias, before BP measurement. First, measure the right radial pulse with a stopwatch for 30 seconds. Then, if the pulse is irregular, bradycardia (less than 30 beats) or tachycardia (more than 50 beats), measure the pulse for 60 seconds to check whether it is regular or not and record it in the relevant column of the questionnaire survey sheet and KDCA web system (classified as normal, abnormal-regular, irregular).

- ◽ Arm position

With the sleeve of the measurement arm fully rolled up, the palm facing up, and the elbow slightly bent. The middle portion of the cuff on the patient’s upper arm is placed at the mid-sternal level or the bottom of the cuff is located 2-3 cm above the elbow using the armrests for heart level. Cuff placement below heart level leads to an overestimation of BP.

- ◽ Cuff selection

The AC determines the cuff size, and the proper size affects the accuracy of BP [24]. A smaller cuff than the required size overestimates BP [25, 26]. Measure the upper AC using a tapeline and select the appropriate cuff size (Table 3).

**Table 3:** Arm circumference and corresponding cuff size for Microlife

**Cuff size of Microlife**	**Cuff width (cm)**	**Cuff length (cm)**	**Arm circumference (cm)**	**Actual** **arm circumference range (cm)**
Child Small (XS)^a^	11	35	14 - 22	14-21.9
Adult Medium (M)^a^	15	53.5	22 - 32	22-31.9
Adult Large (L)^a^	16.5	63.5	32 - 42	32-41.9
Adult Large/Extra Large (L/XL)	16.5	70	32 - 52	42-52

*Microlife* Microlife WatchBP Office AFIB^®^

^a^Internationally validated

- ◽ Palpation of the upper arm (brachial) artery

Before applying the cuff, palpate the brachial artery for 5 to 10 seconds to determine the point at which maximum pulsation is felt.

- ◽ Cuff wrapping

Place the cuff on the upper arm with the artery marking arrow pointing downwards. Check that the lower end of the cuff is positioned 2-3 cm above the subject’s antecubital fossa to match the level of the heart. Next, wrap the cuff tightly around the upper arm. At this point, leave some space between the cuff and the arm. The space is just enough for two fingers to fit in. When wrapping the cuff, ensure that the cuff's tip falls within the sizing line that shows that the cuff size is appropriate.

### BP measurement process

**Fig. 3:**
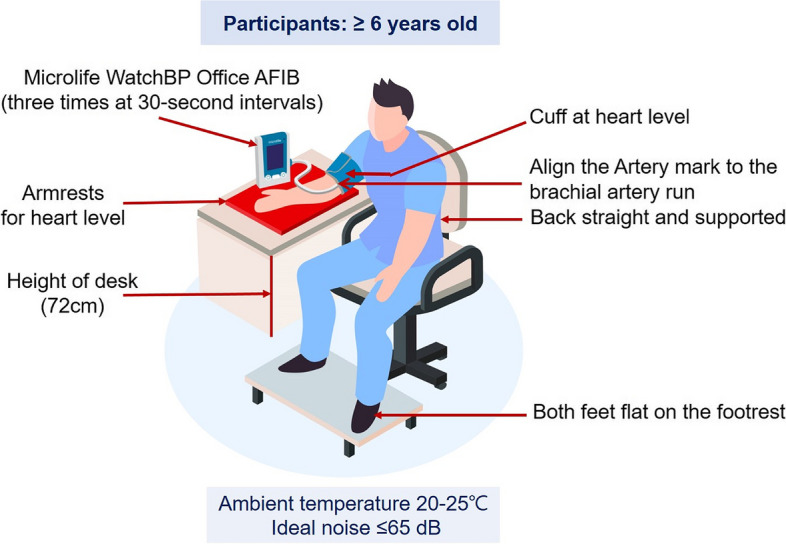
BP measurement using Microlife. BP, blood pressure; Microlife, Microlife WatchBP Office AFIB^®^

**Fig. 4:**
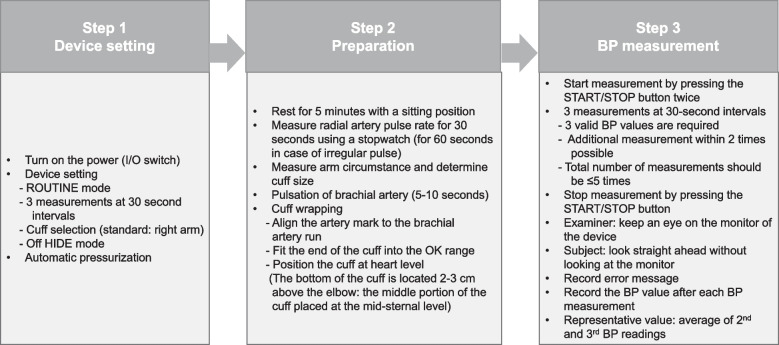
Summary of BP measurement using Microlife. BP, blood pressure. I/O, input/output

Figure 3 shows BP measurement using Microlife. The BP measurement process is as follows:

1. BP and pulse rate measurements should be done before other tests that may affect the results or performance of BP and pulse rate measurements, such as pulmonary function tests and blood sampling. Examiners must be skilled and perform BP measurements in a comfortable and quiet environment.
2. BP measurement process is performed in the presence of an examiner throughout the entire BP measurement process. The examiner closely looks at the monitor of Microlife and checks whether any problems occur, such as measurement error during BP measurement. After stabilization for five minutes, BP is continuously measured to obtain three valid values at 30-second intervals. In order to minimize discomfort and to measure BP within an appropriate time, the additional BP measurement is limited to two times so that the continuous BP measurements do not exceed five times. If less than three BP readings are obtained even after five consecutive measurements, stop the BP measurement and input only the result obtained. In case of an error message, input the error code on the KDCA website and re-measure BP three times.
3. If proper BP measurement is not performed, the device automatically re-measures BP after waiting for 30 seconds.
4. During BP measurement, individual result values ​​should not be viewed by the person being measured to reduce the effect of alarm reaction. This information should be notified in advance before measurement. All steps of BP measurement using Microlife are summarized in Fig. 4.

### Recordings

Each of the following items is entered into a questionnaire survey sheet and KDCA web system: noise, temperature, pulse rate, systolic BP, diastolic BP, the presence of atrial fibrillation, AC, and the use of armrests and footrests.

## Part 2. BP measurement using Greenlight 300^TM^

KNHANES will obtain BP values using Greenlight, as a reference device for future validation studies to replace the MS from 2023.

### Device details

Device structures are shown in Supplementary Figure S1. The Greenlight is a mercury-free auscultatory BP measuring device in which the cuff pressure is displayed as an electronic column using an array of Light Emitting Diodes (LEDs) to replace the mercury column. The Greenlight has been validated based on the ESH-IP [27] and has fulfilled the Universal Standard for validating BP measuring devices [8]. A validation study in Korea provided accuracy comparable to the MS in auscultatory BP measurement within 1 mm Hg error [7]. Components for BP measurement with Greenlight are shown in Supplementary Figure S2.

### Preparations before BP measurement

The BP measurement preparation process is the same as the measurement method using Microlife. Focusing on the functions added to Greenlight, BP measurement methods are demonstrated in Supplementary Figure S3. The upper AC is measured using a tapeline, and the appropriate cuff bladder size of Greenlight is needed according to the AC (Supplementary Table S1).

### BP measurement process

1. Wrap the cuff tightly around the upper arm. The middle portion of the cuff on the patient’s upper arm is placed at the heart level (mid-sternal level) using the armrests. Secure enough space between the bottom of the cuff and the area in contact with the skin of the elbow joint so that the stethoscope can be positioned without touching the cuff.
2. After fully opening the air valve to release pressure, connect the cuff to the Greenlight device using a long connector.
3. Turn on the power by pressing the On/Off switch of the main body. Check that the body is in an auto-zero state and that the pressure is accurately displayed at 0 mmHg on the cuff pressure indicator LEDs. The zero point is automatically adjusted when the power switch is pressed, and the blue light comes on at the zero point.
4. Place the ear insertion part of the stethoscope to face the front and fit snugly into the ear, palpate the upper arm artery, and attach the bell part of the stethoscope to the skin to block noise.
5. Measure the maximum inflation level. While touching the radial artery or the brachial artery, rapidly inflate the cuff up to 70 mmHg and then gradually increase it by 10 mmHg, remembering the pressure when the pulse is no longer palpable. The maximum inflation pressure is the pressure at the point where the pulse is completely lost plus 30 mm Hg. Deflate the cuff quickly and check that the pulse returns. Check that the zero point is restored by fully opening the air valve.
6. First BP measurement: after rapidly increasing the pressure to the maximum inflation pressure, adjust the valve so that the pressure drops at a constant rate of 2 mmHg/sec and auscultate the Korotkoff sound. While reading the scale of the sphygmomanometer, the Greenlight’s lower right and left lights are monitored side-by-side since the deflation speed indicator lights up in blue when the deflation speed is within 2-3 mm Hg/sec. If the red light on the deflation speed excess warning light on the left side of the device lights up, the deflation speed exceeds 3 mmHg/sec. During BP measurement, check whether the deflation speed is too fast only by the color of the light, and the eyes read the scale of the BP monitor.
7. Listen for systolic and diastolic BP. Systolic BP is set to the Korotkoff Phase I sound, and diastolic BP is set to the Korotkoff Phase V sound. Listen to below 10 mmHg after the last sound to confirm that the sound has completely disappeared. Next, loosen the air control valve to relieve pressure quickly, and then disconnect the cuff and the BP measuring device.
8. Repeat the BP measurement process to measure the second and third BP after resting for 30 seconds. The system automatically calculates the subject’s BP as the average of the second and third BP measures. When finished, press the on/off switch of the main body to turn off the power.

## Part 3. Quality control management: calibration process of each device

QC program included the entire process of BP measurement, including environment, examiners, and BP measuring procedures and equipment [5, 28, 29].

### Proper environment for BP measurement

BP is measured in a comfortable and quiet environment (ideal ambient temperature 20-25˚C, ideal noise ≤65 dB).

### Examiner`s education

Four trained nurses in four MECs should pass the regular “quality control and assurance of BP measurement program” three times a year and the program of “video monitoring of weekly calibration process” once a year.

- ◽ Quality control and assurance of the BP measurement program
  1. BP measurement training program to improve the auscultation technique was carried out in two phases.
    In the first phase, examiners pass the examination using a “Non-mercury Auscultation Training Video” produced by the QC team in 2022 consisting of 10 duplicated readings obtained values using a mercury-free Greenlight device. The test was conducted three times a year for the examiners. Validation criteria are as follows: BP differences of ≤5 mmHg in ≥90% of readings and of ≤10 mmHg in ≥95% of readings (level 1); BP differences of the sum of the absolute values of the systolic and diastolic BP of ≤10 mmHg (level 2); BP difference of ≤10 mmHg in ≥90% of readings for evaluating intra-examiner variability (level 3); Examiners must pass Level I to Level III.
    In the second phase, examiners pass “Hands-on Training with Expert”, which evaluate the mean difference in systolic and diastolic BP compared with the expert in three simultaneous readings using Greenlight. The validation criterion is that the BP difference did not exceed 2 mmHg.
  2. Training on how to use a Microlife and Greenlight device.
  3. Video monitoring of each specific BP measurement procedure using Greenlight once a year.
- ◽ Evaluate video monitoring of the skills of examiners for the weekly calibration process by the QC team once a year
  Video monitoring of the skills of examiners for the weekly calibration process including a weekly pressure accuracy test and cuff leakage test was evaluated using an evaluation table by the QC team once a year.
- ◽ On-site evaluation of BP measurement

The QC team regularly visits four MECs for “On-site evaluation of BP measurement “, and he or she checks the entire process of BP measurements, including environment, procedures of BP measurement, and equipment, three times a year with an on-site checklist.

### Device calibration of Microlife

Two BP devices (Microlife or Greenlight) and one MEC simulator (BP3BTO-T^®^, China) are distributed in each MEC. Device calibration of Microlife should be performed using the “five-step QC process of BP devices”, which includes before-use, in-use, and after-use calibration by a manufacturer’s technician based on the British Hypertension Society protocol [29] and by an examiner in each MEC in order to maintain the pressure accuracy of the devices and cuffs. The new devices should be introduced into MECs after passing the before-use calibration process. Then, when using the same device, the before-use calibration in the next year is replaced by the after-use calibration of the current year. The manufacturer’s technician reports the calibration results to the QC team. It is recommended that Microlife should be replaced with a new one every 5-6 years and Greenlight should be replaced new one if its LED lamp did not function.

**Fig. 5:**
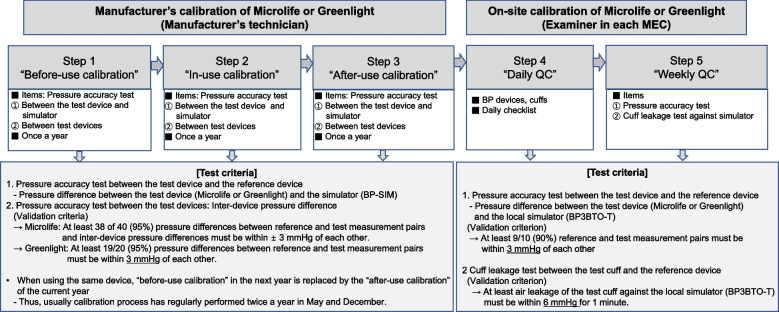
5-Step QC of Microlife and Greenlight. Microlife, Microlife WatchBP Office AFIB^®^; Greenlight, Greenlight 300^TM^; BP-SIM, RIGELBP-BP SIM NIBP simulator^®^; MEC, mobile examination center; QC, quality control

- ◽ Five-step QC process of Microlife
  Five-step QC process of Microlife is summarized in Fig. 5.
  - Step 1 is “before-use calibration” (= pressure accuracy test), performed once a year. This step evaluates the pressure accuracy of the test device against the reference device by the manufacturer’s technicians. RIGELBP-BP SIM NIBP simulator^®^ (BP-SIM, Rigel Medical, USA) [30] was used for reference standard of device validation. The technician takes a picture of the pressure values to determine the accurate pressure difference between the test device and the reference device for each test scale. Four Microlifes and BP-SIM were connected in parallel and obtained 40 readings per device over a range of pressure on 280-60 mmHg scales (10 calls per deflation, 280, 240, 200, 180, 160, 140, 120, 100, 80, 60 mmHg) and calculated the pressure differences between the test device and the reference simulator or between the test devices with multiple comparisons [Supplementary Figure S4 (A)]. Pressure differences between the test device and the reference simulator were calculated by subtracting the test pressure from the reference pressure value for each data point. Then, inter-device pressure differences between Microlifes were calculated. As validation criteria, at least 38 of 40 (95%) pressure differences between the reference and the test measurement pairs and inter-device pressure differences must be within ± 3 mmHg of each other.
  - Step 2 is “in-use calibration”, performed using the same methods as before-use calibration.
  - Step 3 is “after-use calibration”, performed using the same methods as before-use calibration.
  - Step 4 is “daily QC”, carried out by a trained nurse in each MEC with a daily checklist.
  - Step 5 is “weekly QC”. An examiner in each MEC performed a pressure accuracy and cuff leakage test using the MEC simulator. Microlife is placed in the CHECK mode by pressing the START/STOP and I/O switch buttons simultaneously, and the MEC simulator sets ‘SEL 1’ for the pressure accuracy test. The test device (Microlife), air tank, and the MEC simulator is connected with a t-tube [(Supplementary Figure S5 (A)] and obtained 10 pressure readings per device over a range of pressure on 280-60 mmHg scales (10 calls per deflation, 280, 240, 200, 180, 160, 140, 120, 100, 80, 60 mmHg) and calculated the pressure differences. Pressure differences between the MEC simulator and the test devices were calculated by subtracting the test pressure from the reference pressure value for each data point. The validation criteria are that at least 9 of 10 (90%) pressure differences between the reference and the test measurement pairs must be within ± 3 mmHg of each other. The weekly pressure accuracy test is performed once per two weeks. For the cuff leakage test, the MEC simulator sets ‘SEL 2’. After wrapping the cuff tightly around the air tank, the test cuff of Microlife and MEC simulator were connected with a connecting tube [Supplementary Figure S6 (A)] and checked the air leakage at 300 mmHg for one minute and calculated the leakage pressure of the test cuff compared with the MEC simulator. The validation criterion is that leakage pressure must be within ± 6 mmHg in a minute

### Device calibration of Greenlight

The QC process of Greenlight also should be performed similarly to the 5 Steps of Microlife (Fig. 5).

- ◽ 5-Step QC process of Greenlight
  - Step 1 is “before-use calibration” (= pressure accuracy test), performed once a year. Two Greenlight and BP-SIM were connected in parallel [Supplementary Figure S4 (B)] and obtained 20 readings per device over a range of pressure on 280-60 mmHg scales and calculated the pressure differences between the test device and simulator. The validation criterion is that at least 19 of 20 (95%) pressure differences between the reference and test measurement pairs must be within ± 3 mmHg of each other.
  - Step 2 is “in-use calibration”, performed the same methods as “before-use calibration”.
  - Step 3 is “after-use calibration”, performed the same methods as “before-use calibration”.
  - Step 4 is “daily QC”, carried out by an examiner in each MEC using a daily checklist.
  - Step 5 is “weekly QC”, including pressure accuracy and cuff leakage test, carried out by an examiner in MEC using the MEC simulator. MEC simulator sets ‘SEL 1’ for the pressure accuracy test. Then, the test device (Greenlight), air tank, and MEC simulator is connected to t-tube [Supplementary Figure S5 (B)], and the pressure accuracy test is performed similarly to the weekly pressure accuracy test of Microlife. The validation criterion is that at least 9 of 10 (90%) reference and test measurement pairs must be within ± 3 mmHg of each other. The weekly cuff leakage test of the Ambidex^®^ cuff of Greenlight against the MEC simulator is performed using the same methods as the weekly cuff leakage test of the Microlife cuff against the MEC simulator [Supplementary Figure S6 (B)]. The validation criterion is that leakage pressure must be within ± 6 mmHg in a minute.

### Device calibration of the MEC simulator

The MEC simulator (BP3BTO-T^®^) should be performed a pressure accuracy test using a reference device (BP-SIM) once a year (Supplementary Figure S4 (C), Supplementary Figure S7). Two devices are connected and obtained 20 readings per device over a range of pressure on 280-60 mmHg scales and calculated the pressure differences. The Pressure difference between the reference simulator and the MEC simulator was calculated by subtracting the MEC simulator value from the BP-SIM value for each data point. The validation criterion is that at least 19 of 20 (95%) reference simulator and test measurement pairs must be within ± 1 mmHg.

## Conclusions

Accurate BP measurement is crucial for diagnosing and treating hypertension. KNHANES, a representative health survey in Korea, has used MSs for BP measurement since 1998. Due to the MS ban, it now employs Microlife, an oscillometric device, for BP measurement, while using Greenlight as a reference device for validation from 2023. This paper describes the new standardized protocol for BP measurements using Microlife and Greenlight devices in KNHANES, along with their QC process and device calibration. By adopting this standardized protocol and rigorous QC program, KNHANES aims to generate accurate and reliable BP data, thereby ensuring the credibility and efficacy of epidemiological research and public health policymaking in South Korea.

### Supplementary Information

**Additional file 1: Supplementary Table S1.** Arm circumference and corresponding Ambidex cuff size for Greenlight. **Supplementary Figure S1.** Description of Greenlight. **Supplementary Figure S2.** Components of Greenlight. **Supplementary Figure S3.** Summary of BP measurement using Greenlight. **Supplementary Figure S4.** Manufacturer’s pressure accuracy test for Microlife (A), Greenlight (B), and MEC simulator (BP3BTO-T) in Step 1, Step 2, and Step 3. **Supplementary Figure S5.** Pressure accuracy test for Microlife (A) and Greenlight (B) in weekly QC (Step 5). **Supplementary Figure S6.** Cuff leakage test for Microlife (A) and Greenlight (B) in weekly QC (Step 5). **Supplementary Figure S7.** Calibration of MEC simulator.

## Data Availability

Not applicable.

## Abbreviations

BP: blood pressure
KHNANES: Korea National Health and Nutrition Examination Survey
KDCA: Korea Disease Control and Prevention Agency
MSs: Mercury sphygmomanometers
MECs: Mobile examination centers
QC: Quality control
Ads: Auscultatory devices
ODs: Oscillometric devices
Greenlight: Greenlight 300^TM^
Microlife: Microlife, Microlife WatchBP Office AFIB^®^
AC: Arm circumference
ESH-IP: European Society of Hypertension-International Protocol
LEDs: Light Emitting Diodes
BP-SIM: RIGELBP-BP SIM NIBP simulator^®^
