# Supplementary material for: Standardized protocol of blood pressure measurement and quality control program for the Korea National Health and Nutrition Examination Survey
Source: Clin Hypertens. 2023 Oct 12;29:28. doi: 10.1186/s40885-023-00252-7 (PMC10568909; doi:10.1186/s40885-023-00252-7)
Supplement: Supplementary file 1 — Additional file 1: Supplementary Table S1. Arm circumference and corresponding Ambidex cuff size for Greenlight. Supplementary Figure S1. Description of Greenlight. Supplementary Figure S2. Components of Greenlight. Supplementary Figure S3. Summary of BP measurement using Greenlight. Supplementary Figure S4. Manufacturer’s pressure accuracy test for Microlife (A), Greenlight (B), and MEC simulator (BP3BTO-T) in Step 1, Step 2, and Step 3. Supplementary Figure S5. Pressure accuracy test for Microlife (A) and Greenlight (B) in weekly QC (Step 5). Supplementary Figure S6. Cuff leakage test for Microlife (A) and Greenlight (B) in weekly QC (Step 5). Supplementary Figure S7. Calibration of MEC simulator. [file 40885_2023_252_MOESM1_ESM.docx]

**Supplementary data**

**Supplementary Table S1. Arm circumference and corresponding Ambidex cuff size for Greenlight**

| **Cuff size of Ambidex** | **Bladder width**  **(cm)** | **Bladder** **length**  **(cm)** | **Arm circumference (cm)** | **Actual**  **arm circumference range (cm)** |
| --- | --- | --- | --- | --- |
| Infant | 7.0 | 11.2 | 13-19 | 13-17.9 |
| Child | 9.5 | 17 | 18-25 | 18-23.9 |
| Adult | 13.9 | 28.1 | 24-35 | 24-33.9 |
| Large Adult | 15.4 | 32.4 | 34-46 | 34-46 |

Greenlight, Greenlight 300^TM^

**Supplementary Figure S1. Description of Greenlight**


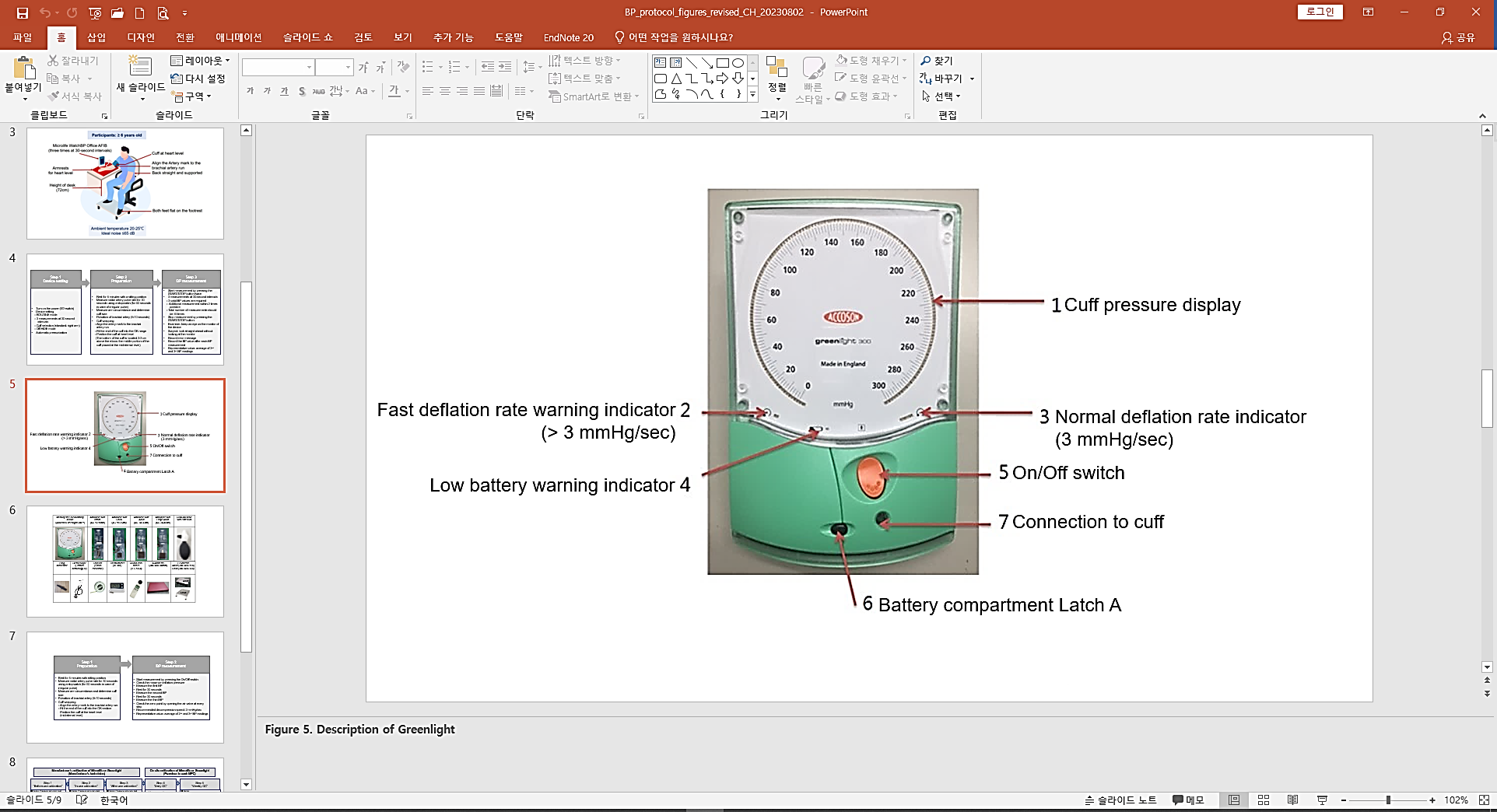


**Supplementary Figure S2. Components of Greenlight**


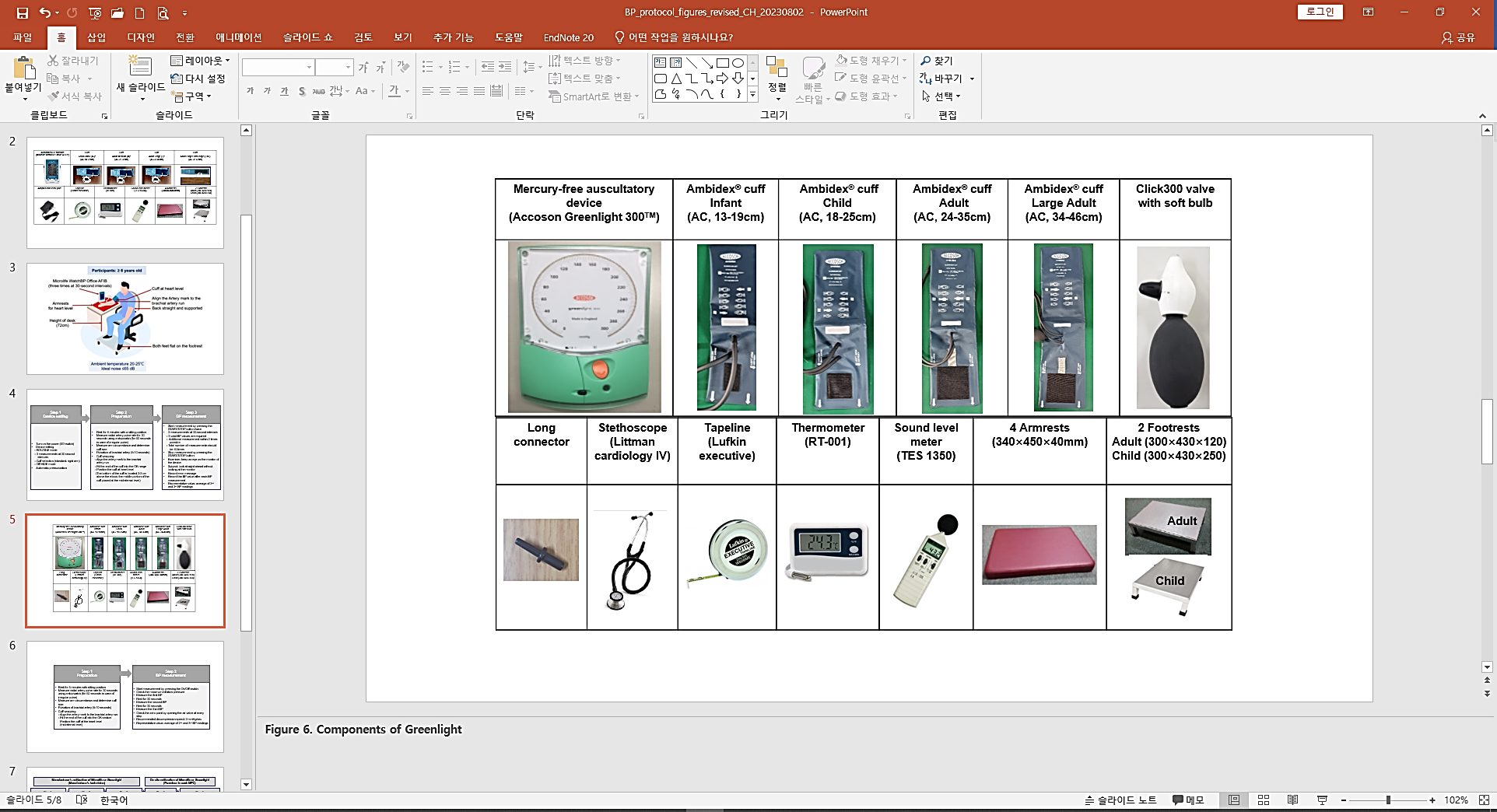


AC, arm circumference.

**Supplementary Figure S3. Summary of BP measurement using Greenlight**


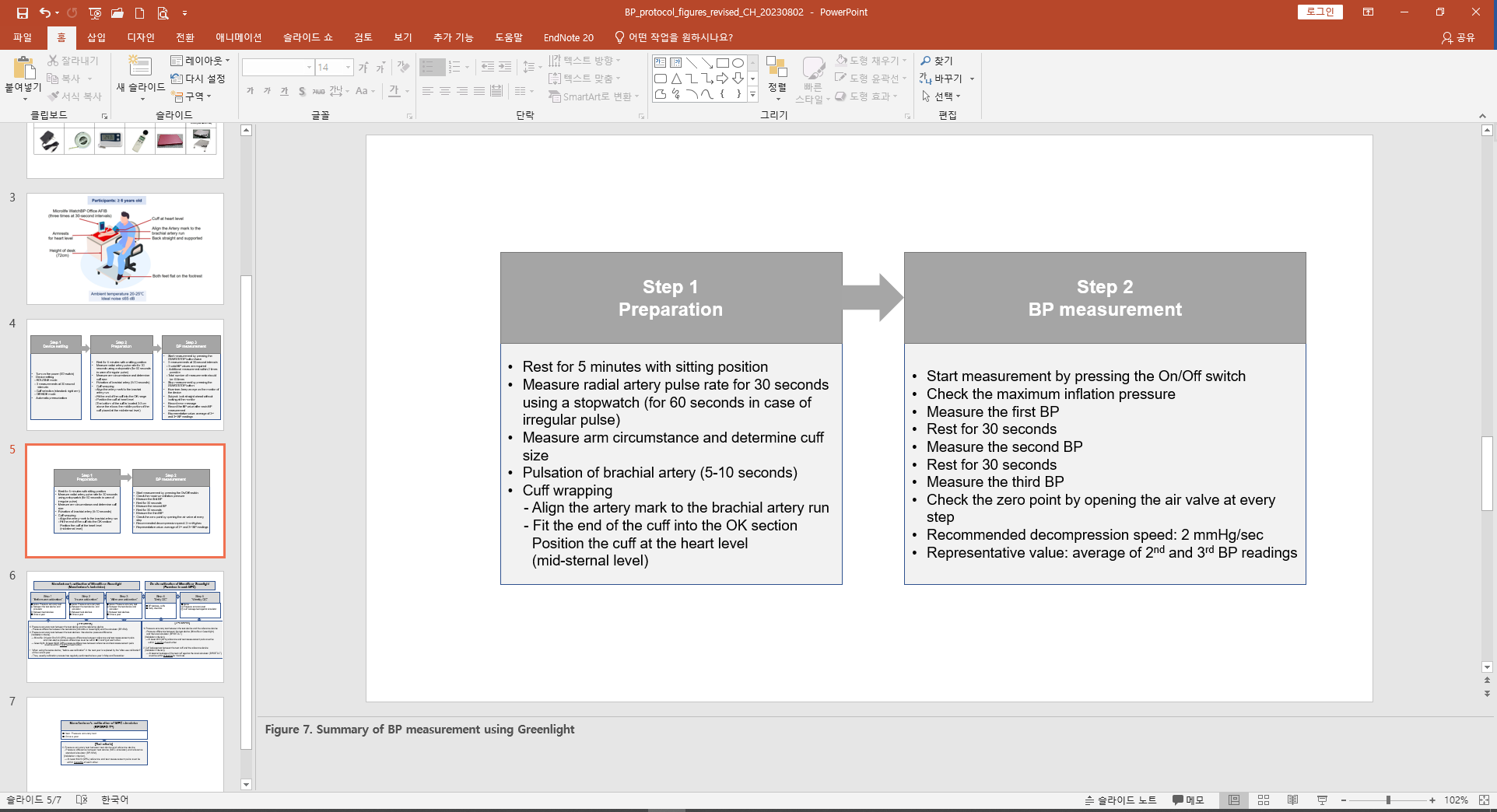


BP, blood pressure.

**Supplementary Figure S4. Manufacturer’s pressure accuracy test for Microlife (A), Greenlight (B), and MEC simulator (BP3BTO-T)** **in Step 1, Step 2, and Step 3**


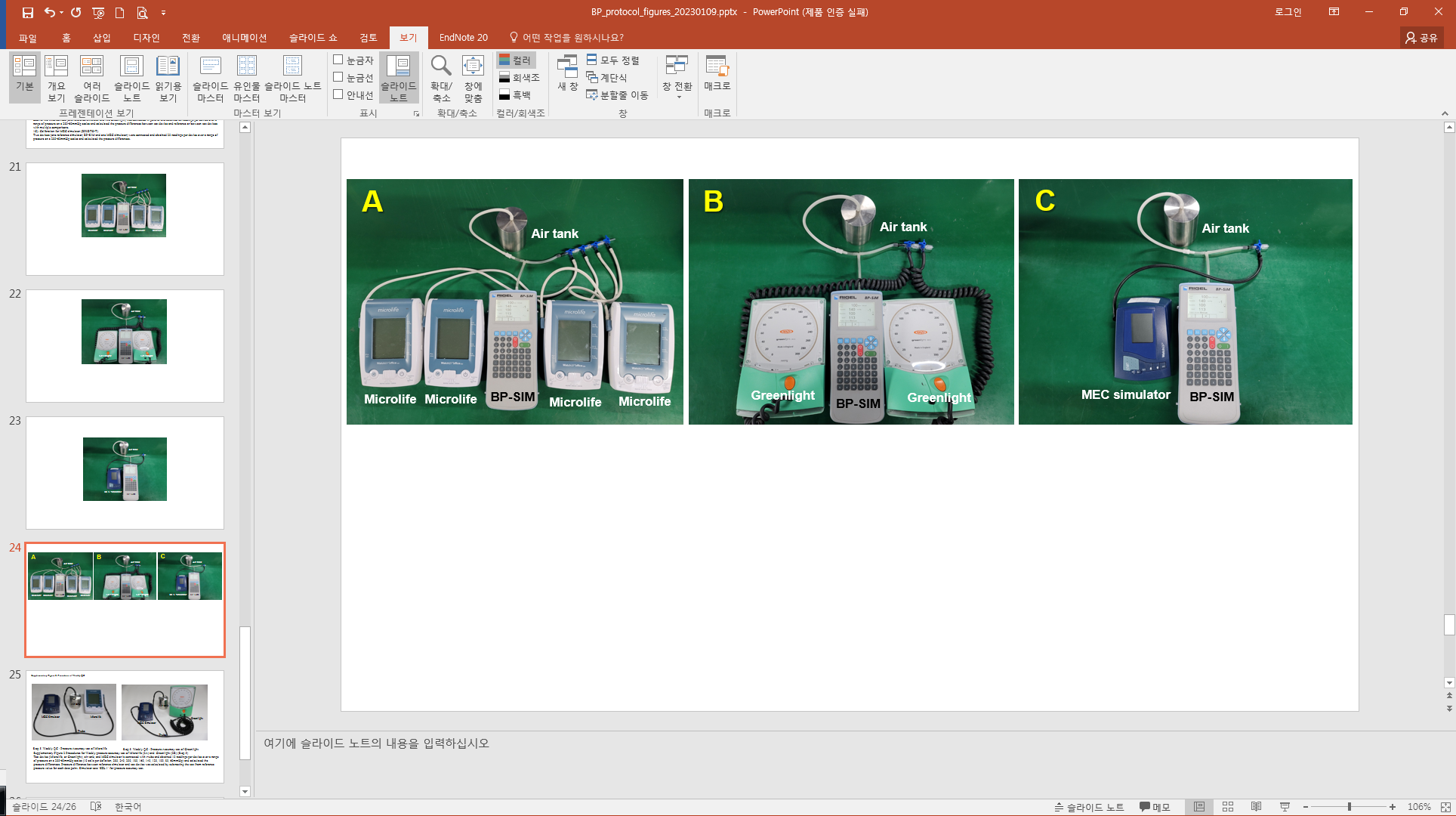


Microlife, Microlife WatchBP Office AFIB^®^; Greenlight, Greenlight 300^TM^; MEC, mobile examination center; BP-SIM, RIGELBP-BP SIM NIBP simulator^®^.

**Supplementary Figure S5. Pressure accuracy test for Microlife (A) and Greenlight (B) in weekly QC (Step 5)**


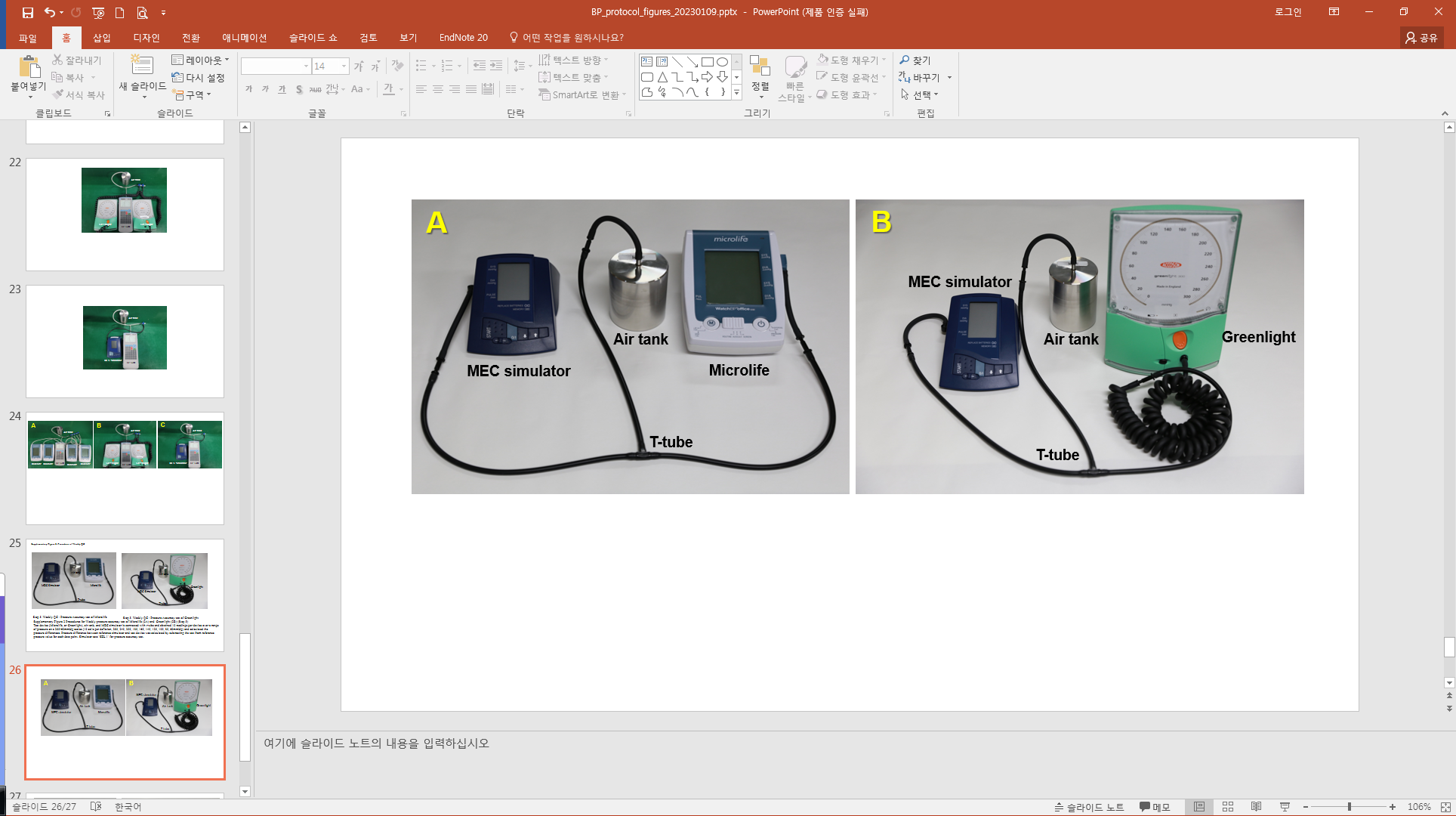


QC, quality control; Microlife, Microlife WatchBP Office AFIB^®^; Greenlight, Greenlight 300^TM^; MEC, mobile examination center.

**Supplementary Figure S6. Cuff leakage test for Microlife (A) and Greenlight (B) in weekly QC (Step 5)**


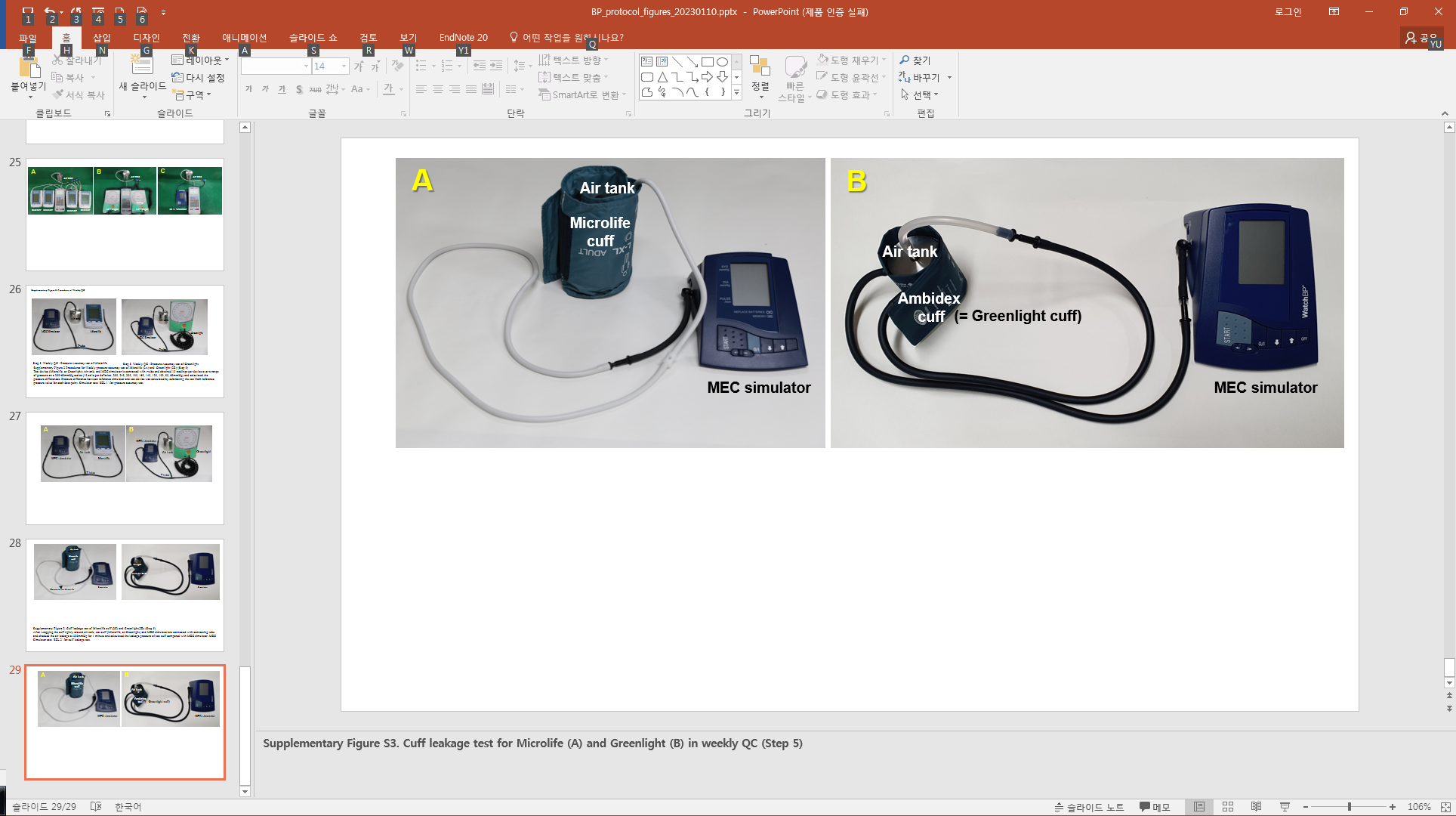


QC, quality control; Microlife, Microlife WatchBP Office AFIB^®^; Greenlight, Greenlight 300^TM®^; MEC, mobile examination center.

**Supplementary Figure S7. Calibration of MEC simulator**


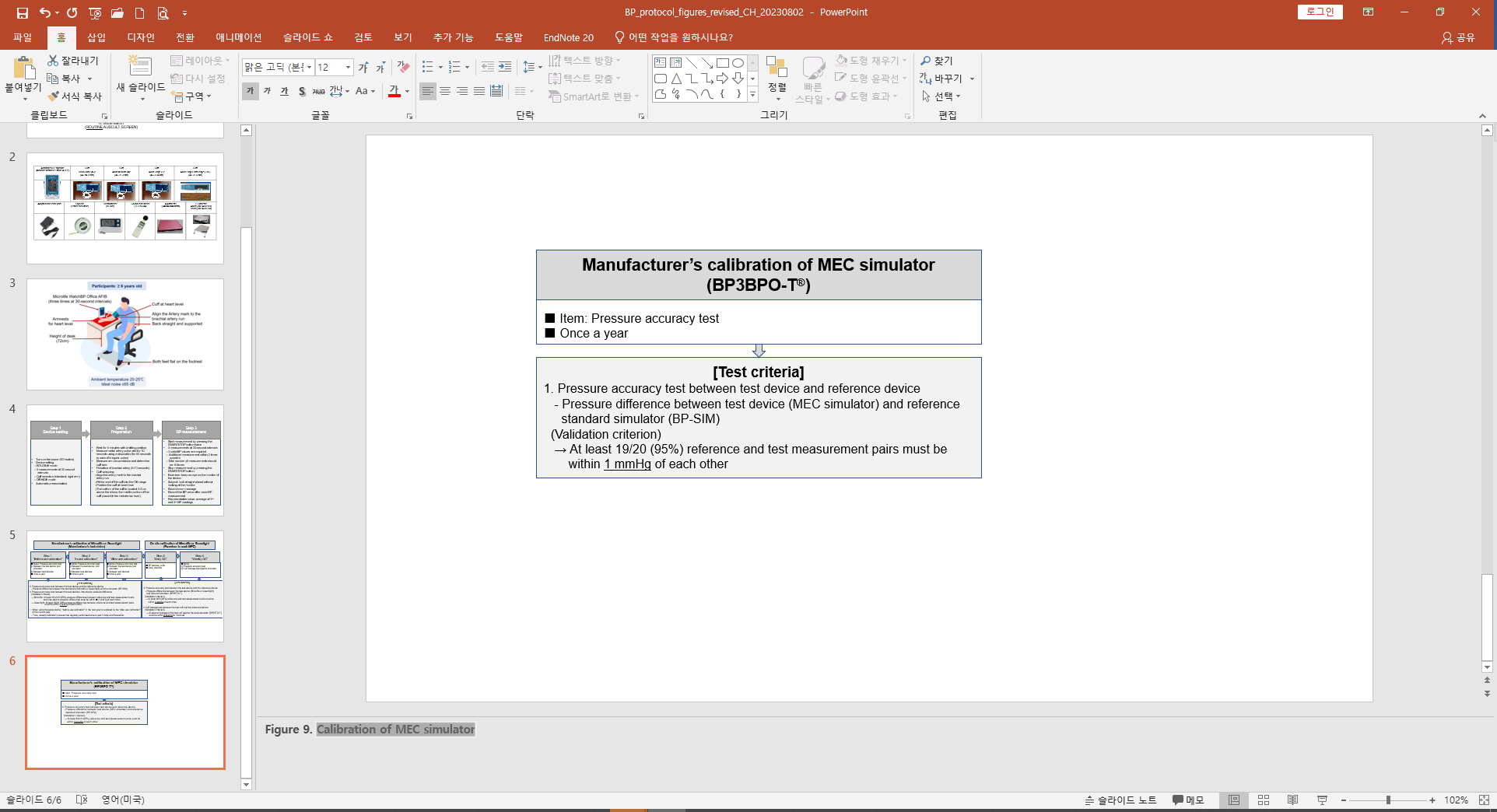


MEC**,** mobile examination center; BP-SIM, RIGELBP-BP SIM NIBP simulator^®^.
